# Ethnic difference in the prevalence of pre-diabetes and diabetes mellitus in regions with Sami and non-Sami populations in Norway – the SAMINOR1 study

**DOI:** 10.3402/ijch.v75.31697

**Published:** 2016-08-08

**Authors:** Ali Naseribafrouei, Bent-Martin Eliassen, Marita Melhus, Ann Ragnhild Broderstad

**Affiliations:** 1 Centre for Sami Health Research, Department of Community Medicine, Faculty of Health Sciences, UiT The Arctic University of Norway, Tromsø, Norway; 2 Department of Medicine, University Hospital of Northern Norway, Harstad, Norway

**Keywords:** prevalence, dysglycaemia, indigenous people, SAMINOR

## Abstract

**Objective:**

The aim of this study was to measure the prevalence of pre-diabetes and diabetes mellitus in rural populations of Norway, as well as to explore potential ethnic disparities with respect to dysglycaemia in Sami and non-Sami populations.

**Design:**

Cross-sectional population-based study.

**Methods:**

The SAMINOR1 study was performed in 2003–2004. The study took place in regions with both Sami and non-Sami populations and had a response rate of 60.9%. Information in the SAMINOR1 study was collected using two self-administered questionnaires, clinical examination and laboratory tests. The present analysis included 15,208 men and women aged 36–79 years from the SAMINOR1 study.

**Results:**

Age-standardised prevalence of pre-diabetes and diabetes mellitus among Sami men was 3.4 and 5.5%, respectively. Corresponding values for non-Sami men were 3.3 and 4.6%. Age-standardised prevalence of pre-diabetes and diabetes mellitus for Sami women was 2.7 and 4.8%, respectively, while corresponding values for non-Sami women were 2.3 and 4.5%. Relative risk ratios for dysglycaemia among Sami participants compared with non-Sami participants were significantly different in different geographical regions, with the southern region having the highest prevalence of pre-diabetes and diabetes mellitus among Sami participants.

**Conclusion:**

We observed a heterogeneity in the prevalence of pre-diabetes and diabetes mellitus in different geographical regions both within and between different ethnic groups.

Norway is home to many ethnic groups, including Norwegians, Kvens and Sami. Kvens are descendants of Finnish ethnicity who immigrated to and settled in the northern parts of Norway in the 1700s and 1800s (1). The Sami people are an indigenous population inhabiting the northern parts of Norway, Sweden, Finland and the Kola Peninsula in Russia. The traditional Sami settlements in Norway span from Finnmark in the north to Engerdal in the Hedmark county in the south. The Sami population harbours a rich variety of languages, cultures and other social circumstances. However, the process of industrialisation has introduced changes in their lifestyle and living conditions. Today, many of the Sami have a sedentary lifestyle, which predisposes them to obesity and type 2 diabetes mellitus (2,3).

Diabetes mellitus is a chronic disease with long-term complications. These complications have become a major cause of morbidity and mortality worldwide and are predicted to increase further in the coming decades (4). The prevalence of diabetes in rural areas has increased to an alarming level in both low- to middle-income countries and high-income countries during the past few decades (5). The estimated prevalence of self-reported diabetes mellitus in people aged ≥30 years in Norway was 3.4% in 2004 (6). The Nord-Trøndelag Health Survey (HUNT) reported a prevalence of diagnosed cases of adult diabetes of 4.3% in 2006 (7). The prevalence of diabetes mellitus and impaired glucose tolerance among the indigenous people of Greenland, the Inuit, has also increased (8).

We lack up-to-date knowledge about the prevalence of pre-diabetes and diabetes mellitus among the inhabitants of northern and mid-Norway, especially regarding eventual ethnic differences. The aim of this study was to measure the prevalence of pre-diabetes and diabetes mellitus in rural populations of Norway, as well as to explore potential ethnic disparities with respect to dysglycaemia in Sami and non-Sami populations.

## Methods

### The SAMINOR1 study

In 2003–2004, the Centre for Sami Health Research at the University of Tromso (UiT) The Arctic University of Norway, in collaboration with the Norwegian Institute of Public Health, conducted the SAMINOR1 study, the first population-based study on health and living conditions in regions with both Sami and Norwegian populations (9). This survey included municipalities and districts in Norway with a high proportion of people with Sami ethnicity, as determined by ethnicity and language information reported in the 1970 census and historical and local knowledge about traditional Sami settlements. These municipalities and districts were almost all located in rural areas. All residents aged 30 and 36–79 years registered in the National Registry in the selected regions were invited to participate in the SAMINOR1 study, regardless of their ethnic background (n = 27,987). Each study participant completed two self-administered questionnaires, which were provided in Norwegian and the three main Sami languages. The clinical investigation was done in two buses moving from place to place throughout the study area. Non-fasting blood samples were taken to determine plasma glucose levels. The Regional Committee for Medical Research Ethics approved the SAMINOR1 study, and all participants gave informed written consent.

### Data collection

The SAMINOR1 study collected information through questionnaires, physical examinations, including anthropometric measures and blood pressure, and blood sampling. The questionnaires covered topics such as language and ethnicity; use of health services and the satisfaction with these services; socio-economic factors; accidents; discrimination; self-reported diseases and illnesses; diseases in the family; mental health symptoms; medication; some questions on diet, smoking, alcohol, physical activity and social networks; and for women only, questions on menstruation, fertility and use of exogenous hormones. Ethnicity was determined through questions such as: “What language(s) do/did you, your parents and your grandparents use at home?”; “What is your, your father's and your mother's ethnic background?”. The respondents were also asked whether they considered themselves to be Norwegian, Sami, Kven or other. The respondents could answer “Sami”, “Norwegian”, “Kven” or “other”. Participants could tick more than one answer for all questions mentioned above. Participants were categorised as Sami if they responded that they either considered themselves to be Sami or reported to have a Sami ethnic background, and if at least one of their grandparents, parents or they themselves spoke a Sami language at home. All other participants were categorised as non-Sami.

Both questionnaire information and non-fasting plasma glucose measurements were used to ascertain the presence of pre-diabetes and diabetes mellitus. Those who reported in the questionnaire that they currently have or previously had diabetes mellitus were classified as having diabetes. In addition, we used a random, non-fasting plasma glucose measurement as an objective method for diagnosing dysglycaemia. Participants with non-fasting plasma glucose levels of ≥11.1 mmol/L were also classified as having diabetes, and those with a level of 7.8–11.0 mmol/L were classified as having pre-diabetes. The remaining participants were categorised as normoglycaemic.

### Geographical regions

**Fig. 1:**
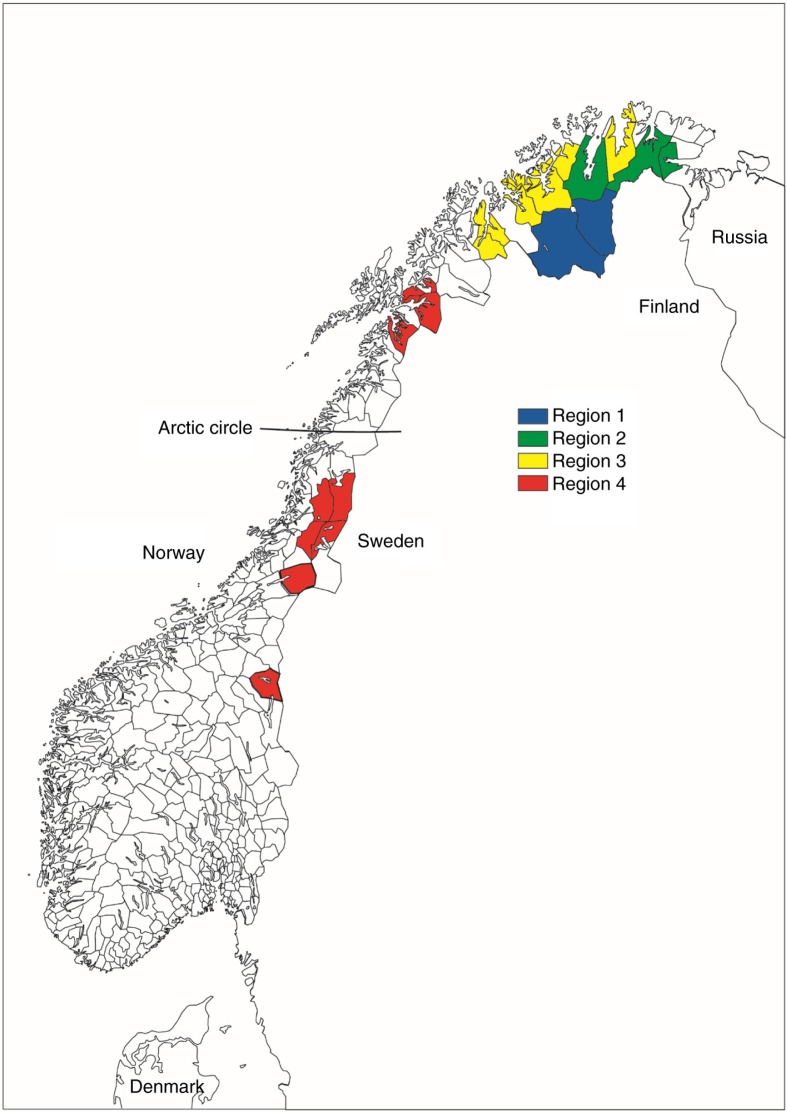
The four geographical regions and the municipalities in each region.

We defined four geographical regions: “Region 1” consisted of areas in the inland of Finnmark county, including Karasjok and Kautokeino municipalities; “Region 2” consisted of both inland and coastal areas in Finnmark county, including Porsanger, Tana and Nesseby municipalities; “Region 3” consisted of coastal areas in Finnmark and the northern part of Troms county, including Lyngen, Storfjord, Kåfjord, Kvænangen, Alta, Loppa, Kvalsund and Lebesby municipalities; “Region 4” consisted of Marka, Lule and south Sami areas in southern Troms, Nordland, Nord- and Sør-Trøndelag counties, including Lavangen, Narvik, Evenes, Skånland, Tysfjord, Hattfjelldal, Røyrvik, Namsskogan, Grane, Snåsa and Røros municipalities (Fig. 1).

### Statistical analysis

The data management and statistical analysis were performed using STATA version 14.1 (StataCorp, College Station, TX, USA). Age difference between ethnic groups across regions and genders was assessed using two sample t-tests (Table II). Education level was not included in the final model as it was not a significant confounding factor and had many missing values. Variables which were strongly correlated to diabetes and/or were parts of metabolic syndrome such as hypertension, dyslipidaemia, obesity and family history of diabetes mellitus were not included in the final regression analysis to avoid overadjustment. Although the questionnaire contained several questions related to lifestyle and socio-economic status, we decided not to include them in the final analysis. The answers to these questions were neither precise nor objective. Furthermore, these factors may have altered since the onset of the disease. There were also many missing values in these variables which could have reduced the statistical strength. The direct method was applied to age-standardise the prevalence of pre-diabetes and diabetes mellitus using the European standard population of 2013 (10). Total prevalence of pre-diabetes and diabetes mellitus for each sex and ethnic group was adjusted according to regional differences in working sample rates. To achieve these adjusted values, the regional prevalence was weighted inversely proportional to the corresponding final working sample percentages (Table I). Multinomial logistic regression stratified by gender and the four geographical regions was used to evaluate age-adjusted relationship between ethnicity (main predictor) and dysglycaemia (outcome). The measure of association is presented as relative risk ratio (rrr=*exp*(*β*)), where *β* is the beta coefficient of the ethnicity variable in the multinomial logistic regression model.

**Table I:** Number of the invitees, participation rates and final working sample in each geographic region by sex (The SAMINOR1 study 2003–2004)

	Invited	Total participation (%)	Working sample (%)
Men	14,114	7,985 (56.6)	7,315 (51.8)
Region 1	1,419	840 (59.2)	528 (37.2)
Region 2	2,202	1,307 (59.4)	1,063 (48.3)
Region 3	7,293	4,186 (57.4)	4,108 (56.3)
Region 4	3,200	1,652 (51.6)	1,616 (50.5)
Women	13,037	8,553 (65.6)	7,893 (60.5)
Region 1	1,285	937 (72.9)	641 (49.9)
Region 2	1,972	1,380 (70.0)	1,158 (58.7)
Region 3	6,785	4,461 (65.7)	4,357 (64.2)
Region 4	2,995	1,775 (59.3)	1,737 (58.0)

Note: We excluded those with unknown ethnicity or ethnicity other than Sami, Norwegian or Kven and those with unknown plasma glucose values from the working sample.

## Results

### Study sample

Due to a low participation rate among 30-year-olds, they were excluded from the study, leaving 27,151 invitees aged 36–79 years. Of these, 16,538 (60.9%) agreed to participate and gave consent to medical research. Participants who reported their ethnic and linguistic background to be other than Sami, Norwegian or Kven or who had missing answers to these questions were excluded (n = 511), as were those with missing plasma glucose levels (n = 819). Thus, 15,208 participants were finally included in the present analysis (Table I).

Of the 15,208 participants included in the study sample, 696 (4.6%) were defined as having diabetes mellitus and 426 (2.8%) as having pre-diabetes. Among those defined as having diabetes mellitus, 636 (91.4%) reported diabetes in the questionnaire, whereas 60 (8.6%) were diagnosed only by non-fasting plasma glucose (data not shown). Table II shows age distribution of participants of both ethnic groups.

**Table II:** Age distribution of the participants by sex, ethnicity and geographical region (the SAMINOR1 study 2003–2004)

Age (years)^a^	n	Sami	n	Non-Sami	p
Men					
Region 1	458	53.3 (52.4–54.3)	70	53.2 (50.7–55.8)	0.94
Region 2	478	55.6 (54.6–56.6)	585	53.8 (52.0–54.6)	0.005
Region 3	541	55.6 (54.7–56.6)	3,567	54.4 (54.1–54.8)	0.017
Region 4	193	54.8 (53.3–56.4)	1,423	56.4 (55.9–57.0)	0.063
Total	1,670	54.9 (54.4–55.4)	5,645	54.8 (54.6–55.1)	0.82
Women					
Region 1	554	53.1 (52.2–54.1)	87	52.4 (50.1–54.7)	0.58
Region 2	504	54.1 (53.2–55.1)	654	53.6 (52.7–54.4)	0.39
Region 3	489	54.7 (53.7–55.8)	3,868	54.2 (53.8–54.5)	0.30
Region 4	181	54.8 (53.1–56.5)	1,556	55.8 (55.3–56.4)	0.26
Total	1,728	54.0 (53.5–54.6)	6,165	54.5 (54.2–54.8)	0.14

Values are mean in years with 95% confidence interval (in parenthesis).

a Tested by two sample *t*-tests with equal variances.

Little or no ethnic difference was seen in the total age-standardised prevalence of pre-diabetes and diabetes mellitus in either sex. Total age-standardised prevalence of pre-diabetes and diabetes mellitus for Sami men was 3.4 and 5.5%, respectively. Corresponding values for non-Sami men was 3.3 and 4.6%. Total age-standardised prevalence of pre-diabetes and diabetes mellitus for Sami women was 2.7% and 4.8%, respectively, while corresponding values for non-Sami women were 2.3 and 4.5% (Table III). In both ethnic groups, the prevalence of pre-diabetes and diabetes mellitus increased considerably with age.

**Table III:** Prevalence of pre-diabetes and diabetes mellitus by sex, age and ethnic group (the SAMINOR1 study, 2003–2004)

Men							
	
Sami				Non-Sami			
		
Age (years)	n	Pre-diabetes	Diabetes	n	Pre-diabetes	Diabetes	p^a^
36–49	576	10 (1.7%)	10 (1.7%)	1,957	41 (2.1%)	29 (1.5%)	0.79
50–59	552	20 (3.6%)	29 (5.2%)	1,776	60 (3.4%)	73 (4.1%)	0.49
60–79	542	26 (4.8%)	51 (9.4%)	1,912	87 (4.5%)	154 (8.0%)	0.57
Total crude	1,670	56 (3.3%)	90 (5.4%)	5,645	188 (3.3%)	256 (4.5%)	0.35
Total age-standardised^b^ (95% CI)		3.4% (2.5–4.2%)	5.5% (4.4–6.6%)		3.3% (2.9–3.8%)	4.6% (4.1–5.2%)	

Women							
	
	Sami			Non-Sami			
		
Age (years)	n	Pre-diabetes	Diabetes	n	Pre-diabetes	Diabetes	

36–49	687	11 (1.6%)	10 (1.7%)	2,272	24 (1.1%)	38 (1.7%)	0.41
50–59	521	16 (3.1%)	15 (2.9%)	1,832	44 (2.4%)	78 (4.3%)	0.26
60–79	520	18 (3.5%)	49 (9.4%)	2,061	69 (3.3%)	156 (7.6%)	0.37
Total crude	1,728	45 (2.6%)	78 (4.3%)	6,165	137 (2.2%)	272 (4.4%)	0.63
Total age-standardised^a^ (95% CI)		2.7% (1.9–3.4%)	4.8% (3.7–5.9%)		2.3% (1.9–2.6%)	4.5% (4.0–5.1%)	

a p-values show the significance level in Pearson's chi-square test

b Direct standardisation using European standard population of 2013 as reference.

CI, confidence interval.

Both Sami men and women had their highest prevalence of pre-diabetes and diabetes in Region 4. While non-Sami men had their highest prevalence of pre-diabetes and diabetes in Region 1, non-Sami women had their lowest prevalence of diabetes in this region (Table IV).

**Table IV:** Crude regional and total prevalence of pre-diabetes and diabetes mellitus, together with total prevalence adjusted for regional working sample by sex, geographical region and ethnic group (the SAMINOR1 study, 2003–2004)

Men						

	Sami			Non-Sami		
		
	n	Pre-diabetes	Diabetes	n	Pre-diabetes	Diabetes
Region 1	458	12 (2.6%)	13 (2.8%)	70	3 (4.3%)	6 (8.6%)
Region 2	478	13 (2.7%)	24 (5.0%)	585	14 (2.4%)	22 (3.8%)
Region 3	541	19 (3.5%)	35 (6.5%)	3,567	191 (3.3%)	171 (4.8%)
Region 4	193	12 (6.2%)	18 (9.3%)	1,423	52 (3.6%)	57 (4.0%)
Crude total	1,670	56 (3.3%)	90 (5.4%)	5,645	188 (3.3%)	256 (4.5%)
Region-adjusted prevalence^a^		3.6%	5.7%		4.4%	4.4%

Women						

	Sami			Non-Sami		
		
	n	Pre-diabetes	Diabetes	n	Pre-diabetes	Diabetes

Region 1	554	15 (2.7%)	29 (5.2%)	87	2 (2.3%)	2 (2.3%)
Region 2	504	10 (2.0%)	12 (2.4%)	654	18 (2.7%)	31 (4.7%)
Region 3	489	12 (2.4%)	23 (4.7%)	3,868	71 (1.8%)	181 (4.7%)
Region 4	181	8 (4.4%)	14 (7.7%)	1,556	46 (3.0%)	58 (3.7%)
Crude total	1,728	45 (2.6%)	78 (4.5%)	6,165	137 (2.2%)	272 (4.4%)
Region-adjusted prevalence^a^		2.8%	4.8%		2.2%	4.3%

a Weighted according to regional working sample rates (see Table I).

In Region 1, the relative risk of having diabetes was significantly lower among Sami men than among non-Sami men (rrr = 0.29) after adjustment for age. The same was observed for Sami women in Region 2 (rrr = 0.46). In Region 4, the situation was reversed, with a relative risk for diabetes mellitus that was significantly higher (rrr = 2.87 for men and rrr = 2.38 for women) in both Sami men and women than in their non-Sami counterparts. Relative risk for pre-diabetes was also significantly higher for Sami men compared with non-Sami men in this region (rrr = 2.05) (Table V).

**Table V:** Age-adjusted relative risk ratios (rrr^a^) for pre-diabetes and diabetes mellitus for Sami compared to non-Sami participants in different regions (the SAMINOR1 study 2003–2004)

		Pre-diabetes			Diabetes		
			
	n	rrr	p	95% CI	rrr	p	95% CI
Men							
Region 1							
	526	0.56	0.38	0.15–2.04	0.29	0.02	0.10–0.82
Region 2							
	1,059	1.12	0.76	0.52–2.42	1.24	0.48	0.68–2.26
Region 3							
	4,104	1.03	0.90	0.63–1.69	1.29	0.20	0.87–1.88
Region 4							
	1,610	2.05	0.03	1.06–3.96	2.87	0.00	1.63–5.06
Women							
Region 1							
	638	1.18	0.82	0.26–5.30	2.23	0.28	0.52–9.64
Region 2							
	1,155	0.68	0.33	0.31–1.49	0.46	0.03	0.23–0.91
Region 3							
	4,337	1.31	0.39	0.70–2.43	0.97	0.91	0.62–1.53
Region 4							
	1,733	1.63	0.21	0.75–3.53	2.38	0.01	1.28–4.43

a The measure of association is presented as relative risk ratio (rrr) = exp(β), where β is the beta coefficient of the ethnicity variable in the multinomial logistic regression model. 95% CI, 95% confidence interval.

## Discussion

In this study, we found statistically significant differences in the relative risk of diabetes mellitus between the Sami and non-Sami populations in some geographical regions. While the odds of having diabetes were lower for Sami men in Region 1 and Sami women in Region 2, the opposite was seen in the southern region, where the Sami were more prone to diabetes mellitus. Except for men in Region 4, prevalence of pre-diabetes was not significantly different between the Sami and non-Sami populations.

Two other studies based on data from the SAMINOR1 study have focused on diabetes prevalence. Nystad in her PhD showed no difference in the prevalence of type 2 diabetes mellitus between Sami and non-Sami populations (11). However, the definition of Sami ethnicity in Nystad's study focused more on linguistic features. Moreover, that study considered only self-reported diabetes and did not take into account regional differences. It is worth mentioning that if we merged participants of the same ethnicity from all the geographical regions we considered, there would be no statistically significant difference between the two ethnic groups. Broderstad and Melhus showed that although there was no ethnic difference in the prevalence of diabetes, ethnicity appeared to affect the type of diabetes treatment (12).

The HUNT3 study was conducted in 2006 in North Trøndelag county in the middle part of Norway and reported a prevalence of known (i.e. previously diagnosed) diabetes mellitus of 4.9 and 3.9%, respectively, in men and women aged ≥20 years. However, the prevalence of undiagnosed type 2 diabetes mellitus was estimated to be as high as that of known type 2 diabetes (7). However, considering the higher age of our participants (≥36), our use of non-fasting plasma glucose to diagnose diabetes mellitus and the heterogeneity of the prevalence of diabetes mellitus in the different geographical regions, it would be challenging to compare the results. In a follow-up study of the first Finnmark study (1974–1975), it was established that Sami women were more obese but did not have a higher incidence of diabetes mellitus than other women (13). Our findings were similar to that of the Finnmark study, which indicated that Sami women had higher truncal obesity (results not shown) but not a significantly higher rate of pre-diabetes or diabetes mellitus. In another study recently conducted in Greenland, the prevalence of type 2 diabetes among the Inuit was estimated around 9%, of which 79% were previously unknown cases (14). In a cross-sectional study, the prevalence of diabetes mellitus varied among the three Alaskan Inuit populations, with the Siberian Yupik (9.6%) having the highest rates, followed by the Central Yupik (2.8%) and Inupiat participants (3.7%). In the Alaskan study, diabetes was more prevalent in women than in men (8.8% vs. 4.2%), and of the people identified with diabetes in the study, 47% had not been previously diagnosed (15).

In contrast to these studies, the prevalence of undiagnosed diabetes mellitus was not so high in our study (8.6%). This may be the result of an effective and affordable health system in Norway, with sufficient coverage in rural areas with indigenous inhabitants. Another explanation for this may be the low sensitivity of non-fasting blood glucose to diagnose diabetes mellitus.

In 2004, the estimated sex- and age-standardised prevalence of known diabetes mellitus among those aged ≥30 years in Norway was 3.4% (6). Although this prevalence was lower than ours, the age composition of participants and the methods applied to diagnose diabetes mellitus were rather different from ours, making it difficult to compare the results. In 2002, the prevalence of diabetes among people aged 45–64 years in Iceland was reported to be 4.9% in men and 2.9% in women, reflecting an increase of around 50% over a period of 30 years (16). Previous estimates of age- and sex-specific prevalence of known diabetes mellitus in Denmark, Finland and Sweden are also comparable to our results (17–19).

In our study, we compared the prevalence of pre-diabetes and diabetes mellitus between the Sami and non-Sami and found a heterogeneity across sexes and geographical regions. The four geographical regions that we considered in our study all have their own characteristic features such as location, climate, majority or minority status of the Sami population, implementation of preservation measures for Sami language, dialect, diet and religion.

In Region 1, Inland in Finnmark County, the Sami comprise 80–90% of the population (9), and some of the most important Sami-related institutions, such as the Sami Parliament and Sami University College (Sámi allaskuvla), are located there. Reindeer husbandry is more prevalent here than in other regions; hence, it is quite natural that reindeer is a large part of the diet of the inhabitants (2). In this region, Sami men had significantly lower prevalence of diabetes mellitus. Although Sami women were more obese than their non-Sami counterparts, no significant differences were observed in their prevalence of pre-diabetes or diabetes mellitus.

In Region 2, the Sami account for about half of the population (9). The municipalities in this region have both coastal and inland regions, with many farmers, fishermen and reindeer herders. The prevalence of diabetes in this region was significantly lower in Sami women than their non-Sami counterparts.

Region 3 represents a traditional coastal Sami population. Assimilation policies (Norwegianisation process) had a huge effect in these coastal regions (20), and in most of these municipalities, the Sami are now a minority. We found no ethnic difference in pre-diabetes or diabetes mellitus prevalence in this region.

Region 4 has a more heterogeneous population than the other regions. Three distinct Sami groups inhabit this region: the Marka Sami, Lule Sami and South Sami. Each has their own Sami language. By the second half of the 19th century, the Sami languages were already in retreat in this region (21). The proportion of the population with Sami ethnicity is lower in this region than in any of the other geographical regions we investigated (9). The prevalence of diabetes mellitus among the Sami in this region was more than twice as high as that among the non-Sami population. It is not clear which factor is responsible for this high prevalence. However, one interesting common feature observed in the groups with the highest prevalence of diabetes mellitus (the Sami in Region 4 and the non-Sami in Regions 1 and 2) was that they lived in a minority setting. Further studies need to be performed to clarify this phenomenon.

### Strength and limitations

A relatively high participation rate (60.9%) and large sample size (15,208) in 24 municipalities made it possible for us to perform an in-depth analysis of diabetes status and related explanatory variables. As opposed to former studies on the prevalence of dysglycaemia, we were able to take into account the difference between geographical regions from which participants were recruited and heterogeneity across ethnic groups.

In our analysis, definition of the Sami was based on whether participants self-identified as Sami or had a Sami ethnic background, and if they, their parents or grandparents spoke Sami. This definition is rather different from the definitions of Sami used in the Finnmark study, “Ung i Nord” (The North Norwegian Youth Study) or former publications from the SAMINOR1 study, which used language as a basis. We chose to emphasise self-identification, as the Sami language has been subject to discrimination and stigmatisation and much of it might have been lost (22). The difference in how Sami ethnicity was defined might make comparison between our results and those from other studies difficult (23).

In this study, we used both self-reported diabetes and non-fasting plasma glucose to ascertain diabetes mellitus status. A non-fasting plasma glucose value of 11.1 mmol/L (200 mg/dl) or greater, together with symptoms, is an established diagnostic criterion for diabetes, but this method is not very reliable. The reliability of this diagnostic criterion is affected by the natural fluctuations of blood glucose throughout the day and can usually only detect diabetes that is poorly controlled (24). By the time this study was performed, HbA1c had not been standardised and approved to be applied for diagnosing diabetes mellitus. The SAMINOR1 study had a large number of participants attending per day, thus it was not feasible to conduct a 2-hour plasma glucose tolerance test. It was furthermore inadvisable to have participants arrive at the medical station after overnight fasting, as the time schedule was distributed during the day. In the present study, we did not perform any medical examination to find signs and symptoms of hyperglycaemia nor did we use other tests such as the glucose tolerance test or fasting plasma glucose to confirm the results of non-fasting plasma glucose tests. Furthermore, the use of self-reported information on diabetes may lead to some uncertainty and misclassification. Indeed, although some studies have proven that questionnaires are a convenient, yet valid, tool for studying chronic diseases such as diabetes and have satisfactory concordance with medical records (25), the validity of the self-administered questionnaire used in the SAMINOR1 study has not yet been determined.

In the present study, we did not distinguish between type 1, type 2 and gestational diabetes due to a lack of information and the need for exhaustive tests. Considering that around 80% of diabetes cases are type 2 diabetes mellitus (26), and given the age of the participants (36–79 years), we assumed that almost all of the cases in our study were of type 2 diabetes mellitus.

The present study had a cross-sectional design, making it difficult to assess potential causal relationships due to temporal bias. We decided not to include physical activity due to the possibility of temporal bias, which might have obscured the relationship between exposure and outcome. Moreover, diabetes or its comorbidities and/or complications might have altered the health-related behaviour and attitudes of those affected. Education was also excluded from the regression analyses as no confounding effect was observed for it. In addition, those risk factors which were part of metabolic syndrome like hypertension, dyslipidaemia and obesity were not included in the regression analysis to avoid overadjustment.

As we stratified the data by sex and region, we reduced the number of participants in each regression analysis and consequently reduced the statistical strength. An uneven distribution of participants from different ethnic groups in different geographical regions exacerbated this problem.

Non-responders tended to be younger, single and male (27), but other than this, there was very limited information, making it difficult to assess potential selection bias. As it was not possible to determine the response rate by ethnicity, it is not possible to attribute the pure burden and differences in the prevalence of pre-diabetes or diabetes mellitus to differences in participation rates. Another limitation of the study is that it was conducted in 2003–2004. Considering the relatively long time since then, caution should be exercised before applying the results to present-day populations.

## Conclusion

The most striking finding in our study was the heterogeneity in the prevalence of pre-diabetes and diabetes mellitus in different geographical regions. While the prevalence of diabetes mellitus was lower in the Sami population of some northern regions, it was much higher in the southern region compared with their non-Sami counterparts. In future, further studies should be performed to address the potential explaining factors behind the observed heterogeneous discrepancies between the prevalence of pre-diabetes and diabetes mellitus in the two ethnic groups. Preventive measures should be implemented at the population level to reduce the levels of established risk factors for developing diabetes, with a special focus on those with pre-diabetes and people living in regions where a higher prevalence of diabetes mellitus has been reported.
